# Dabbing-Induced Hypersensitivity Pneumonitis

**DOI:** 10.7759/cureus.16333

**Published:** 2021-07-12

**Authors:** Ibrahim Haddad, Farah AL-Ghzawi, Sajin M Karakattu, Rasheed Musa, Girendra Hoskere

**Affiliations:** 1 Internal Medicine, East Tennessee State University, Johnson City, USA; 2 Internal Medicine, Hashemite University, Zarqa, JOR; 3 Pulmonary and Critical Care Medicine, East Tennessee State University, Johnson City, USA

**Keywords:** dabbing, tetrahydrocannabinol, hypersensitivity pneumonitis, case report, bho

## Abstract

Dabbing has been gaining popularity among young people in recent years due to its ability to deliver a high concentration of tetrahydrocannabinol. When produced illegally, it is usually contaminated by toxic substances and associated with multiple health hazards. We present the case of a 66-year-old woman who developed hypersensitivity pneumonitis after dabbing butane hash oil for the first time and was successfully treated with corticosteroids with complete resolution of her symptoms. This case report emphasizes the respiratory complications associated with using a noxious substance like butane hash oil and gives physicians an insight into the diagnosis and management of dabbing-induced hypersensitivity pneumonitis.

## Introduction

Dabbing is the process of ingesting high concentrations of tetrahydrocannabinol (THC) through heating and vaporizing butane hash oil (a waxy substance with the street name BHO). This vapor is then inhaled by the user, offering significantly high THC concentrations in a single dose [1]. Based on user reports, dabbing is different from the more common flower cannabis usage by containing a much higher concentration of THC [2,3]. Another distinction of dabbing is the presence of impurities and unpurged butane in the vapors inhaled by the users along with the THC [2]. Due to its perceived ease of production, dabbing popularity is rapidly increasing in recent years among marijuana users as a new method for administering cannabinoids [3].

## Case presentation

**Figure 1:**
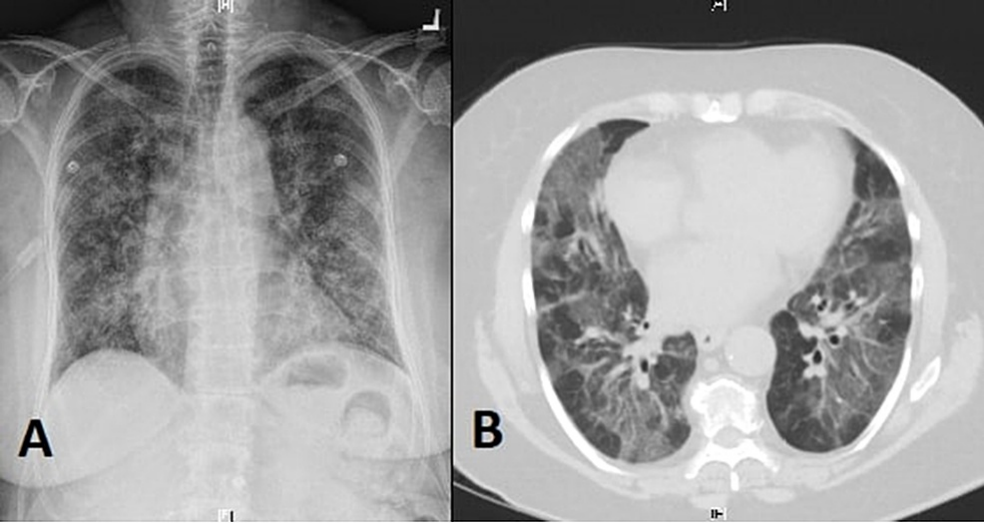
Chest X-ray showing diffuse micronodular pattern with patchy foci (A). CT scan of the chest showing bilateral extensive patchy ground-glass opacity (B).

A 66-year-old female with a past medical history significant for chronic obstructive pulmonary disease (COPD) presented to our hospital with acute-onset shortness of breath and dry cough. She reported trying dabbing BHO for the first time 24 hours before the onset of her symptoms. At presentation, she endorsed the presence of dyspnea and dry cough but denied chest pain, palpitations, hemoptysis, lower-extremities swelling, dizziness, or syncopal episodes. She also denied fever, chills, nausea, vomiting, diarrhea, or abdominal pain. She was tachypneic, tachycardiac, and hypoxic with an O_2_ saturation of 90% on 3 L nasal cannula but afebrile. Physical examination was positive for diffuse pulmonary crackles and scattered wheezes over both lungs' fields but otherwise unremarkable. Labs showed leukocytosis of 14,300/uL. A chest X-ray showed a diffuse micronodular pattern with patchy infiltrates (Figure 1A). A computed tomography (CT) scan of the chest was done and showed extensive patchy ground-glass opacity throughout both lungs' fields, suggestive of inflammatory pneumonitis (Figure 1B). Based on the patient's clinical presentation and the temporal relationship between dabbing and the development of her symptoms, she was diagnosed with hypersensitivity pneumonitis and started on daily 60 mg prednisone for treatment. After three days, her dyspnea and cough improved significantly, and her oxygen requirement went back to normal. A follow-up chest X-ray showed a complete resolution of the infiltrative process. She was discharged home on daily 60 mg prednisone for a total of two weeks, followed by slow tapering with a recommendation to avoid dabbing or any other inhalational cannabinoid usage.

## Discussion

Dabbing utilizes heating and inhaling extracts that are often created using a process that involves butane, hence the term "butane hash oil" (BHO), which is far more potent than flower cannabis [4,5]. The process of producing BHO begins with blasting a liquid butane through a glass tube containing dry cannabis with a filter used at one end of the tube, the filter entraps the large pieces of cannabis but allows the liquid remnants of THC and butane oil to pass into a bowl. After that, the bowl is heated by placing it on a hot surface to allow for the partial evaporation of the butane, leaving behind a waxy brown substance that is then known as BHO [6]. BHO has 75% THC and a concentration of terpenes that ranges from 0.1% to 34% when made illegally, while traditionally smoked cannabis has a THC concentration of 5-20% THC [7].

Dabbing vapor contains multiple toxic substances, including methacrolein (MC) and benzene. MC is structurally similar to acrolein, a noxious pulmonary irritant, and is speculated to cause lung injury and edema in the same mechanism as its related compound [7]. Acrolein is also mutagenic and carcinogenic, and it is hypothesized that it induces these effects through oxidative DNA damage and modification and degradation of DNA repair proteins [8]. On the other hand, benzene is a well-known toxic and carcinogenic substance, and it is considered the most significant single known cancer-risk air toxin [9]. Benzene concentration in dabbing vapor is far higher than that found in ambient air [7,10].

BHO has a high concentration of THC, which increases the risk of lower respiratory tract infection through inducing bronchial ciliary loss and impairing alveolar macrophages and other immunocytes' function [11,12]. Cannabis might be contaminated with fungus and bacteria, and their inhalation has been linked to allergic bronchopulmonary aspergillosis and hypersensitivity pneumonitis, respectively [13]. Although after reviewing the literature, we could not find quantitative data to validate or ascertain the microbial contaminants of BHO. Also, chronic cannabis usage has been linked to increased rates of bronchitis and COPD^ ^[14].

The temporal relationship between BHO exposure and the development of hypersensitivity pneumonitis in our patient is suggestive of a causal relationship. However, the exact underlying pathophysiological mechanism is unclear, but it could be related to direct inhalation injury and maladaptive host immune response induced by butane or other impurities [15]. Imaging findings in cannabis-induced hypersensitivity pneumonitis among synthetic marijuana users include diffuse centrilobular nodules with a tree-in-bud pattern on chest CT scan and a diffuse micronodular pattern with patchy foci on chest X-ray [16]. The treatment approach is similar to other cases of hypersensitivity pneumonitis. It includes avoidance of dabbing or any other inhalational cannabinoid usage and corticosteroid therapy, usually with daily 60 mg prednisone for one to two weeks, followed by slow tapering [17].

## Conclusions

With the rapid increase of dabbing among marijuana users, investigating the problems that users may experience from dabbing becomes increasingly important for the diagnosis of respiratory complications. The lack of literature on dabbing and its widespread use makes the risks and effects on patients and its users unknown. 

Dabbing BHO is associated with acute and chronic pulmonary complications. We recommend that physicians should consider dabbing-induced hypersensitivity pneumonitis as a differential diagnosis for acute pulmonary infiltrate among patients who dab BHO. Also, they should consider the route of administration for patients using marijuana to widen the differential diagnosis. 
